# Endurance versus resistance training in treatment of cardiovascular risk factors: A randomized cross-over trial

**DOI:** 10.1371/journal.pone.0274082

**Published:** 2022-09-06

**Authors:** Hannah J. Thomas, Channa E. Marsh, Barbara A. Maslen, Leanne Lester, Louise H. Naylor, Daniel J. Green

**Affiliations:** School of Human Sciences (Exercise and Sport Sciences), The University of Western Australia, Perth, Western Australia, Australia; Pennington Biomedical Research Center, UNITED STATES

## Abstract

**Background:**

Individual variability in traditional cardiovascular risk factor responses to different exercise modalities has not been directly addressed in humans using a randomized cross-over design.

**Methods:**

Body weight and body mass index, resting blood pressure, blood glucose, insulin and lipids were assessed in 68 healthy untrained adults (26±6 years) who underwent three-months of exercise training targeted at improving cardiopulmonary fitness (endurance) and skeletal muscle function (resistance), separated by three-months washout.

**Results:**

There were significant increases in weight and body mass index following resistance (+0.8 kg, P<0.01; and +0.26 kg/m^2^, P<0.01, respectively), but not endurance (+0.1 kg, P = 0.75; and +0.03 kg/m^2^, P = 0.70, respectively). Although no significant group changes resulted from training in other cardiovascular risk factors, the positive response rate for all variables ranged from 27–49% for resistance and 42–58% for endurance. Between 39–59% of individuals who did not respond to resistance nonetheless responded to endurance, and 28–54% who did not respond to endurance responded to resistance.

**Conclusion:**

Whilst, on average, 12 weeks of resistance or endurance did not change most cardiovascular risk factors, many subjects showed robust positive responses. Exercise modality had an impact on the proportion of subjects who responded to training, and non-response to one mode of training did not imply non-response to the alternate mode. Although the effect of exercise on a single risk factor may be modest, the effect on overall cardiovascular risk profile can be dramatic.

**Study registration:**

The study was registered at the Australian New Zealand Clinical Trials Registry, which was published prior to recruitment and randomization (ACTRN12616001095459).

## Introduction

Promotion of exercise and physical activity is a cornerstone in the prevention and management of cardiovascular (CV) and metabolic diseases [1–3], recommended by professional organizations as a first line strategy to combat the unhealthy consequences of contemporary western lifestyles. There is compelling epidemiological evidence for the beneficial effects of exercise on CV events and mortality [1, 4], supported by studies which have suggested that exercise can have beneficial effects on various CV risk factors [1, 5, 6] and on atherosclerotic progression [1, 4, 7, 8]. Nonetheless, exercise is often perceived as relatively ineffective in modifying traditional CV risk factors in primary care, leading to a reliance on pharmacological strategies. Indeed, some evidence suggested that a proportion of individuals exhibit *adverse* CV risk factor responses to exercise interventions [9]. Assessment of the variability in responsiveness to distinct forms of exercise, when all subjects are supervised, monitored and adherent to a matched prescription, has not previously been characterized.

Research is often focused on the *intensity* of exercise that may be best to improve risk factors related to CV diseases [10–14]. However, different exercise *modalities* can be prescribed to specifically target distinct physiological outcomes, such as enhanced fitness and CV adaptation versus increased skeletal muscle mass and function. Exercise modality is therefore an important parameter to consider when optimizing exercise prescription for individualized health benefit [15–17]. Resistance (RES) and endurance (END) exercise are distinct modalities of training that are commonly utilized in this regard. Despite this, research directly comparing the effects of RES and END training effects on CV risk factors in the same participants is limited. We aimed to investigate 1) whether RES or END training has a greater effect on CV risk factors within individuals; and 2) whether the responder rate and concordance of response for change in CV risk factors differs according to exercise modality. We hypothesized that 1) END would result in a greater reduction in risk factors compared to RES; and 2) individuals who did not respond positively to one form of training would respond to the alternate mode of training.

## Methods

Full details of the study design and experimental procedures can be found in our comprehensive protocol paper [18] and in the study registration (ACTRN12616001095459) at the Australian New Zealand Clinical Trials Registry, which was published prior to recruitment and randomization. Of note, all subjects in this study were pairs of monozygotic and dizygotic twins. For the purpose of this report, we present group and individual responses; heritability analyses are not included. Nonetheless, we have adjusted all analyses for twin correlations (please see statistical analysis section of methods).

### Participants

**Fig 1:**
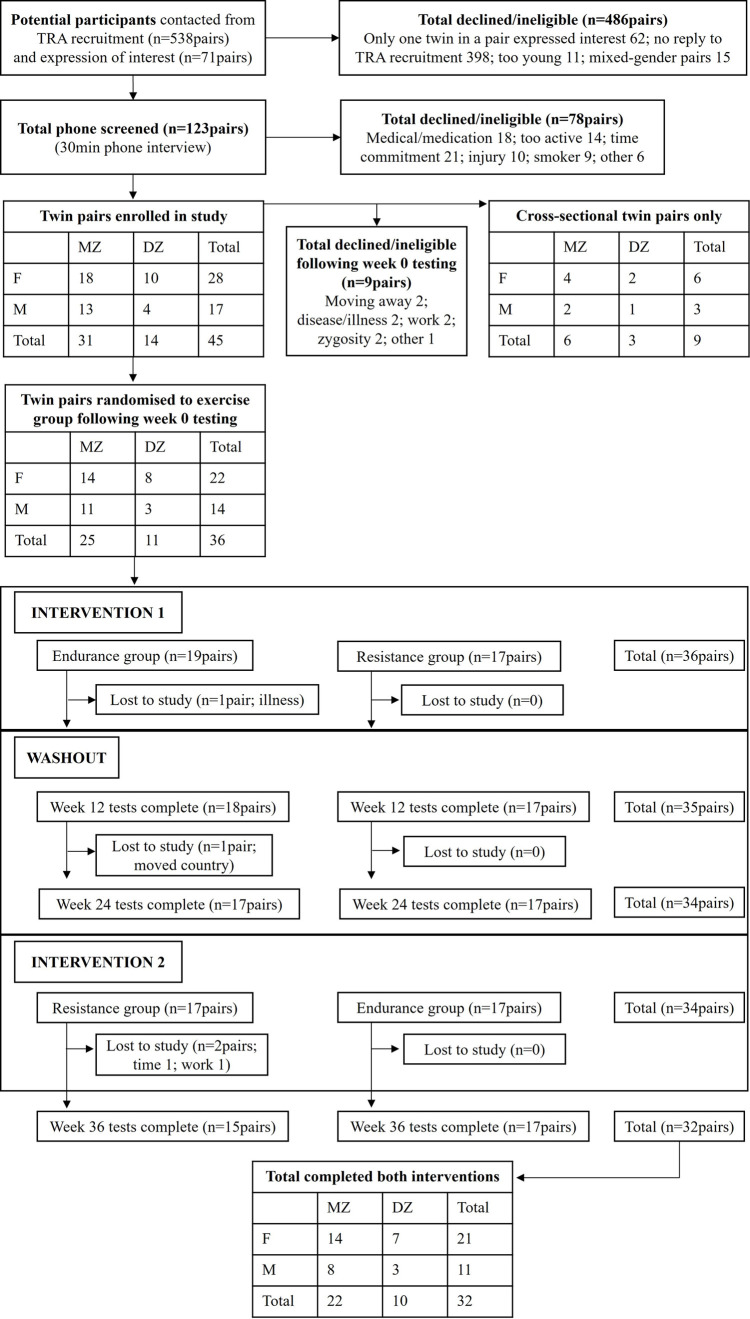
Consort flow diagram.

Sixty-eight healthy young adults (26 ± 6 yrs; 59% female) were recruited to participate in the study (Fig 1). Recruitment included advertising in newspapers, online and via social media, university email lists, and word-of-mouth referral. Inclusion criteria were healthy, relatively unfit individuals who did not meet Australian guidelines for physical activity recommendations (<150min/week), were non-smokers and medication free. This study was approved by the University of Western Australia Human Research Ethics Committee (reference number RA/4/7031). Oral and written informed consent was obtained from all subjects prior to participation in the study, which conformed to the Declaration of Helsinki. Baseline data are displayed in Table 1.

**Table 1:** Baseline characteristics of participants enrolled in the study.

	Baseline (n = 68)
	Female n = 40 and Male n = 28
Age (yrs)	26 ± 6
Height (cm)	173.4 ± 7.2
Weight (kg)	70.3 ± 14.6
BMI (kg/m^2^)	23.3 ± 4.0
WHR	0.76 ± 0.07
Waist girth (cm)	76.2 ± 11.1
Hip girth (cm)	100.1 ± 7.9
SBP (mmHg)	113 ± 9
DBP (mmHg)	64 ± 8
MAP (mmHg)	82 ± 8
Insulin (mU/L)	6.93 ± 3.51
Glucose (mmol/L)	4.67 ± 0.42
HOMA-IR	1.48 ± 0.78
Cholesterol (mmol/L)	4.39 ± 0.77
Triglycerides (mmol/L)	0.96 ± 0.45
LDL (mmol/L)	2.55 ± 0.66
HDL (mmol/L)	1.39 ± 0.32
Chol/HDL	3.32 ± 0.91

BMI = body mass index, WHR = waist to hip ratio, LDL = low-density lipoprotein, HDL = high-density lipoprotein, HOMA-IR = homeostatic model assessment for insulin resistance, SBP = systolic blood pressure, DBP = diastolic blood pressure, MAP = mean arterial pressure. Data are mean ± SD.

### Study design

Outcome measures were collected in all participants within 14 days of commencing and completing each intervention, as detailed below. These measures included anthropometry data [height, weight, body mass index (BMI), waist and hip girth, waist hip ratio (WHR)], resting blood pressure (BP), and blood draws for cholesterol measures, triglycerides, insulin, and glucose. Participants were randomized, using a website-based randomization tool, to three months of either RES or END exercise training, before undergoing a three-month washout period, during which they were instructed to maintain their usual activities and diet. Participants then crossed over to complete three months of their second, alternate, exercise intervention (RES or END). Fat and lean mass data were assessed using dual energy X-ray absorptiometry (DXA). Standard calibration and quality assurance procedures were used (www.gehealthcare.com). Participants arrived at the laboratory at the same time of day for each of the four measurements (before and after each exercise intervention) and were instructed to fast for 3 h before their scan and maintain normal hydration. These data have been reported elsewhere [16]. Fitness data, collected using a graded exercise cardiorespiratory test (VO_2_max), and strength data, assessed using a one-repetition max (1RM) test, pre and post each exercise intervention, has also been reported elsewhere [18].

### Exercise interventions

The center-based, supervised exercise interventions consisted of three one-hour sessions per week for 12 weeks. The programs were intensity matched between volunteers and progressively overloaded across the 12 weeks, consisting of specified training phases. The exercise modalities were not matched for workload or energy expenditure; we opted to assess the impact of ecologically valid exercise prescriptions typical of those used in gymnasia and industry settings. A more detailed explanation of the exercise interventions is provided in our protocol paper [18].

END utilized two running and one cycling session per week, progressing from 60 to 90% VO_2_max_,_ which was monitored via continuous heart rate (HR) using a HR monitor (Polar RS300X HR monitor, Polar Electro Oy, Finland). Target HRs were calculated from HR at a percentage of the initial graded exercise VO_2_max test so that they were individualized, but matched, for intensity between individuals. The 12-week END program was interval based (i.e., walking/running/cycling bouts were interspersed with rest periods), adapting many principles from previously published work [19, 20].

RES alternated between upper and lower body exercises and sessions which progressed from 60 to 90% of 1RM. RES was monitored by recording the number of repetitions, sets and weight completed, as well as a rating of perceived exertion for each set. Individual weights were prescribed from each participant’s pre-training 1RM so that they were individualized, but matched, for intensity between individuals. Each session focused on one of the five main exercises (two upper body–bench press and standing military press; three lower body–squats, deadlift, and leg press), alternating upper and lower body on separate days. There were secondary exercises performed during each session that used other muscle groups (i.e., staggered feet leg press, seated row, lat pulldown). Participants performed a standardized warm up before completing their session and a standardized five minutes of core exercises and cool down at the end of the session. To guide participants’ progressions, 1RM assessments were repeated halfway through their 12-week program.

### Primary outcome measures

#### Anthropometry measures

Participants arrived at the laboratory at the same time each morning for repeated measures following an overnight fast. Participants had refrained from any moderate/vigorous physical activity and alcohol for the 24 h prior to the testing session. This was confirmed at the start of each session verbally and recorded on the testing sheet. These conditions were also adopted for the resting BP and blood draw measures. Height was measured using a fixed and calibrated stadiometer, and body weight was measured on the same scales throughout the study (Tanita Corporation, Tokyo, Japan). BMI was calculated as weight in kg divided by height in meters squared. Hip and waist girth were measured three times with a constant-tension tape measure (Lufkin W606PM Cooper industries SC, USA) with the median value used as the outcome measure. WHR was calculated as waist measurement divided by hip measurement.

#### Resting blood pressure

Supine resting BPs on the right arm were taken every three minutes over a total of 15 minutes using an automated sphygmomanometer (Dinamap V100, GE, Healthcare, USA). This was done in a quiet dark room with the subject alone, undisturbed and lying supine. Mean arterial BP (MAP), systolic BP (SBP) and diastolic BP (DBP) of the last two assessments were recorded and averaged.

#### Blood draws

Blood was drawn from the antecubital fossa using a 21G needle by a trained phlebotomist in the UWA laboratory, into three collection tubes (3X Vacutainer, DB). This was done after the blood pressure assessments described above. Sterile techniques were applied, identical to those used in routine clinical services, and a maximum of 20 mL of blood was drawn per visit. Three blood tubes (2X lithium heparin, 1X fluoride oxalate) were analyzed by a commercial pathology laboratory, for lipids (total cholesterol, triglycerides, HDL, low-density lipoprotein (LDL) and the ratio of total cholesterol and HDL), fasted blood glucose and insulin. Homeostatic model assessment for insulin resistance (HOMA-IR) was calculated as glucose times insulin divided by 22.5.

### Statistical analysis

**Fig 2:**
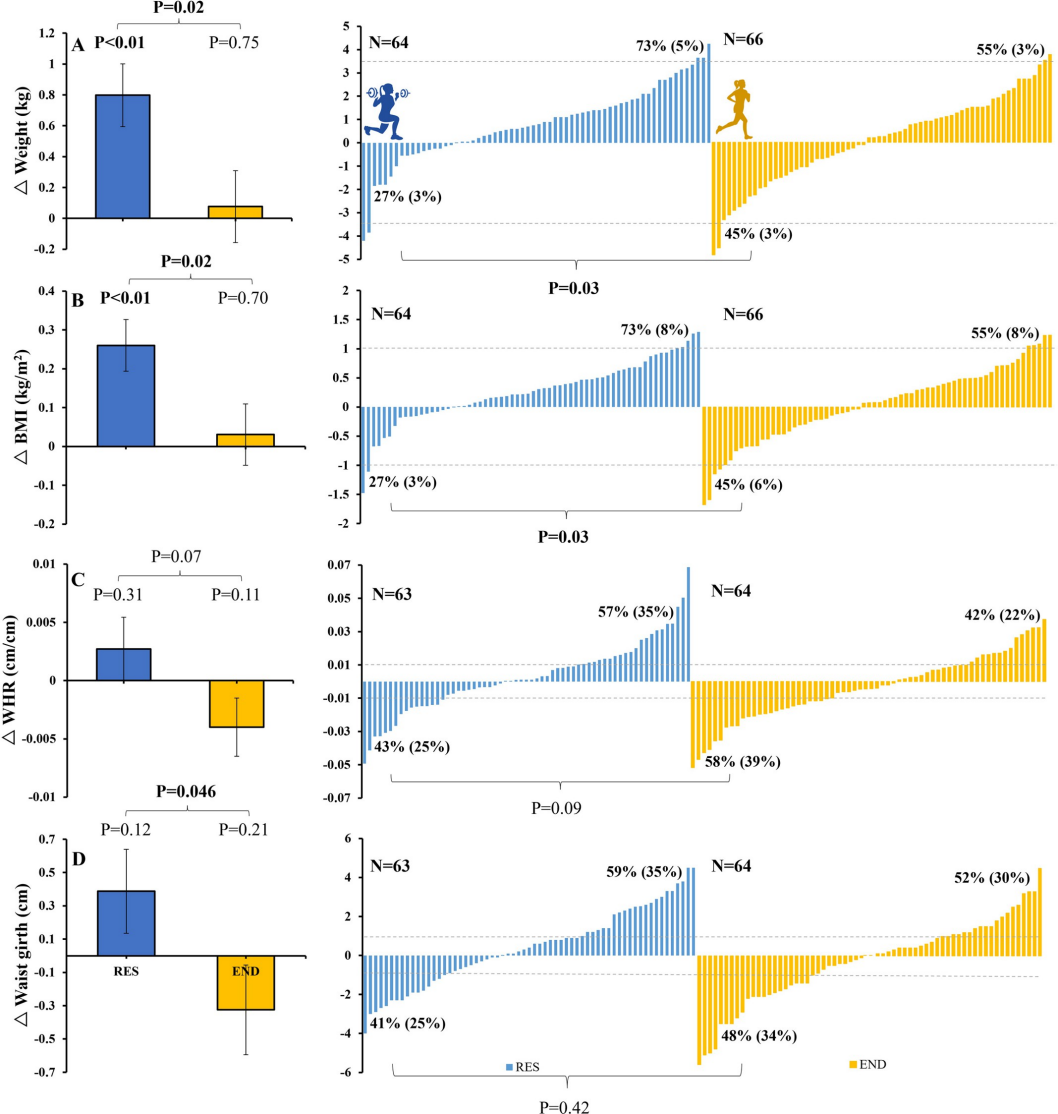
Changes (Δ) with RES (blue) and END (yellow) training for body weight (top panel, A), BMI (panel B), WHR (panel C), and waist girth (panel D) for group (left) and individual responses to RES (middle) and END (right) training. Dotted lines represent previously reported clinically meaningful changes. A 5% decrease in weight (equating to 3.5 kg for our study participants) has been considered to be clinically meaningful [21, 22]. A 1 kg/m^2^ increase in BMI was associated with a 5% (M) and 7% (F) increased risk of heart failure and a 4–6% increased risk of stroke [27]. A 0.01 unit increase in WHR was associated with a 5% increased risk of future CV disease [28]. A 1cm increase in waist girth was associated with a 2% increased risk of future CV disease [28]. Proportions (%) are indicated for responders (any positive change) and non-responders (negative response or no change). Numbers in parentheses reflect responses equal to or beyond the level of clinically meaningful change, as indicated by the dotted lines.

**Fig 3:**
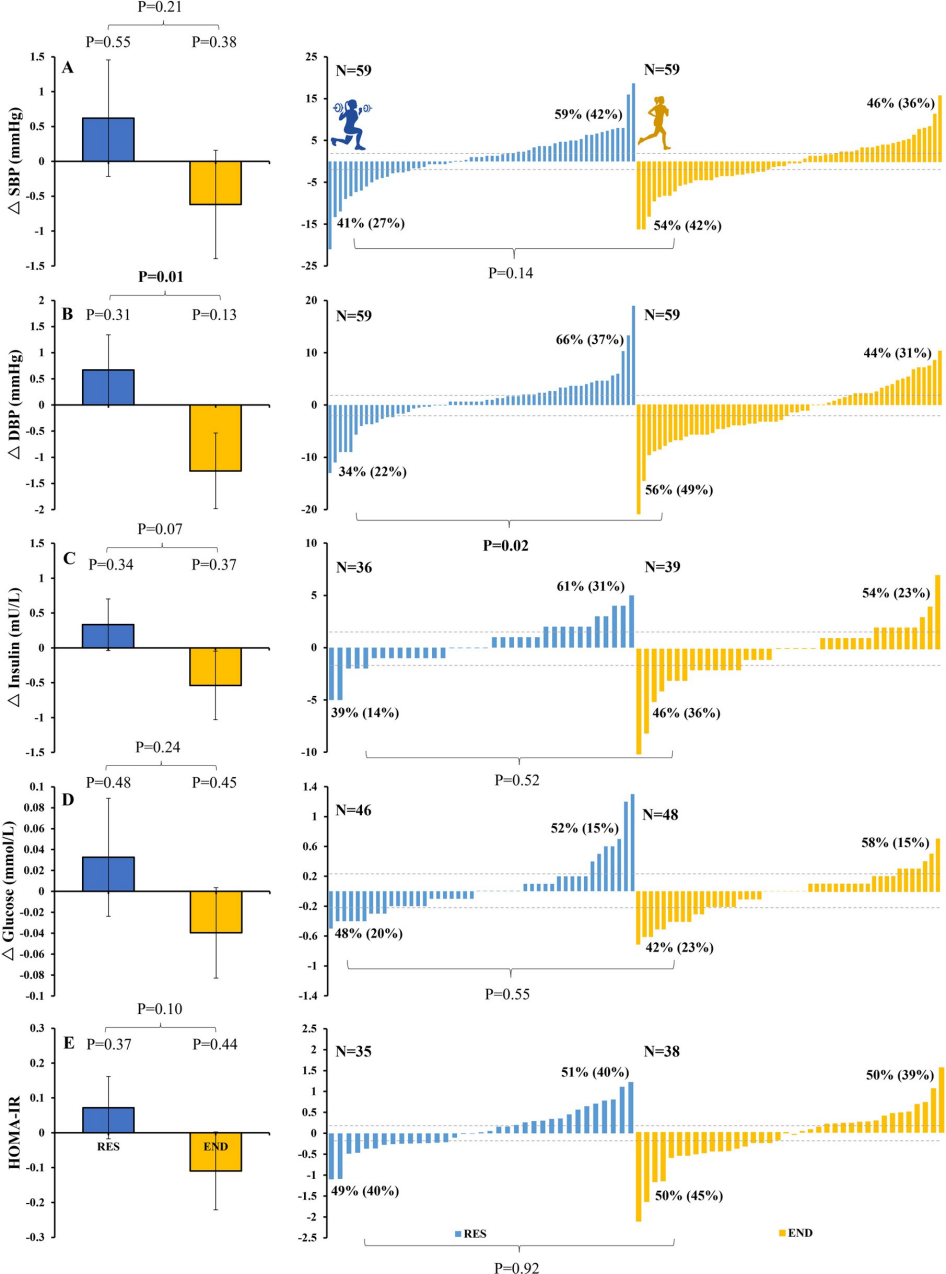
Changes (Δ) with RES (blue) and END (yellow) training for SBP (top panel, A), DBP (panel B), insulin (panel C), glucose (panel D), and HOMA-IR (panel E) for group (left) and individual responses to RES (middle) and END (right) training. Dotted lines represent previously reported clinically meaningful changes. A 2-mmHg decrease in SBP was associated with a 7% lower risk of mortality from ischemic heart disease [23]. A 2-mmHg decrease in DBP was associated with a 12% decreased risk of ischemic heart disease [23]. 0.5SD increase in insulin (equating to 1.73 mU/L for our study participants) was associated with a 6.5% increased risk of CV disease [24]. 0.5SD increase in glucose (equating to 0.21 mmol/L for our study participants) was associated with a 10.5% increased risk of coronary heart disease [24]. 0.25SD increase in HOMA-IR (equating to 0.20 units for our study population) was associated with a 11.5% increased risk of coronary heart disease [24]. Proportions (%) are indicated for responders (any positive change) and non-responders (negative response or no change). Numbers in parentheses reflect responses equal to or beyond the level of clinically meaningful change, as indicated by the dotted lines.

**Fig 4:**
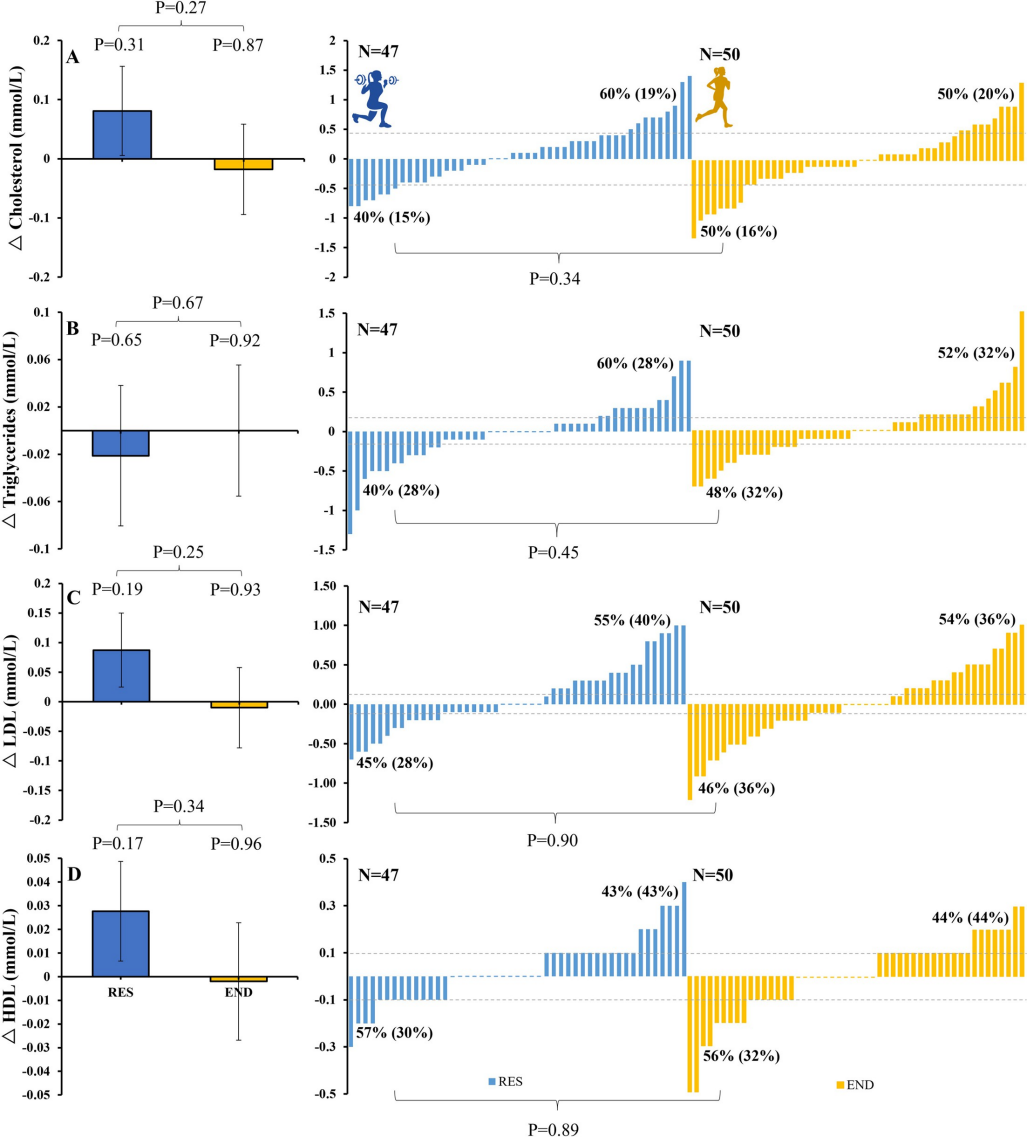
Changes (Δ) with RES (blue) and END (yellow) training for cholesterol (top panel, A), triglycerides (panel B), LDL (panel C), and HDL (panel D) for group (left) and individual responses to RES (middle) and END (right) training. Dotted lines represent previously reported clinically meaningful changes. A 10% decrease in cholesterol (equating to 0.439 mmol/L for our study participants) was associated with a 15% decrease in coronary heart disease mortality [29]. A 19% decrease in triglycerides (equating to 0.182 mmol/L for our study participants) was associated with a 26% decrease in total mortality [26]. A 4% decrease in LDL (equating to 0.102 mmol/L for our study participants) was associated with a 8–12% decrease in the risk of coronary heart disease [25]. A 0.100 mmol/L increase in HDL was associated with a 8–12% decrease in the risk of coronary heart disease [25]. Proportions (%) are indicated for responders (any positive change) and non-responders (negative response or no change). Numbers in parentheses reflect responses equal to or beyond the level of clinically meaningful change, as indicated by the dotted lines.

Statistical analyses were performed with STATA v15 software (StataCorp, College Station, Texas). The effects of the exercise interventions (Figs 2–4) were assessed for each outcome measure using a linear mixed model which accounted for the repeated and paired nature of the data, with age and sex as covariates. An independent covariance structure has been used in the linear mixed models as it allows for a distinct variance for each random effect. The fixed effects of the linear mixed model were specified as regression parameters (time, workload, and age) and the random-effects portion of the model were specified by considering the grouping structure of the data (pairing). Carry-over and order effects were also assessed using linear mixed models adjusting for twin correlations. A Z-test was used to assess the differences between percentages of responders for exercise interventions (RES vs END) for each variable. A responder was simply defined as any positive response in an outcome variable, while a non-responder was defined as a negative response (or no response) in an outcome variable. A Wilcoxon signed-rank test was used to determine significance between increases in the number of risk factors improved when participants undertook END compared to RES (for 21 subjects who possessed data for all 11 risk factors). The proportion of individuals who did not respond to RES but had a positive response from END training was compared to the converse response profile using a Chi-squared test.

#### Power calculation and sample size

Power tests were performed assuming two-tailed tests for 5 target variables, with alpha 0.05, effect sizes estimated on the basis of minimal clinically significant changes for each variable and standard deviations derived from technical errors and coefficients of variation for repeated measure in an exercise training intervention study [9]. For body weight, where minimal clinically significant change was defined as 5% (~3.5kg) [21, 22] and the variability for repeated measures was 0.7kg [9], 68 subjects yield a power >0.99. For SBP, where minimal clinically significant change was defined as a 2-mmHg decrease [23] and the variability for repeated measures was 4.9 mmHg [9], 68 subjects yield a power >0.90. For insulin, where minimal clinically significant change was defined as 0.5SD increase from the mean (~1.76 mU/L) [24] and the variability for repeated measures was 1.73 mU/L [9], 68 subjects yield a power >0.99. For HDL, where minimal clinically significant change was defined as 0.100 mmol/L increase [25] and the variability for repeated measures was 0.06 mmol/L [9], 68 subjects yield a power >0.99. For triglycerides, where minimal clinically significant change was defined as a 19% decrease (~0.182 mmol/L) [26] and the variability for repeated measures was 0.21 mmol/L [9], 68 subjects yield a power >0.99.

## Results

### Participant characteristics

Baseline characteristics for the 68 participants included in this study are provided in Table 1. No adverse events occurred during the course of this study.

### Exercise training—efficacy

Attendance at training sessions was 94% for RES and 95% for END. As previously published [17], VO_2_max significantly increased by 3.61 ± 3.77 mL.kg^-1^.min^-1^ (P<0.01) in response to END (0.25 ± 0.26 L.min^-1^, P<0.01), but not in response to RES (0.03 ± 3.57 mL.kg^-1^.min^-1^ and 0.04 ± 0.25 L.min^-1^). In contrast, 1RM significantly increased in response to RES (leg press: +47.0 ± 29.4 kg, P<0.01, bench press: +5.1 ± 5.0 kg, P<0.01) but not END (leg press: +3.0 ± 26.4 kg, bench press: -0.4 ± 3.4 kg). Both RES and END significantly increased total lean mass (1156 ± 1132 g, P<0.01 and 430 ± 1111 g, P<0.01, respectively) and decreased total fat mass (-351 ± 1403 g, P<0.05 and -495 ± 1464 g, P = 0.006, respectively) [16].

### Impact of RES and END on CV risk factors: Group means

#### Anthropometry data

Group means for anthropometry data in response to RES and END training are shown in Fig 2. In response to RES training both weight (Δ 0.80 ± 1.63 g, P<0.01) and BMI (Δ 0.26 ± 0.53, P<0.01) significantly increased, while there was no significant change in WHR, waist or hip girth. In response to END training there was no change in weight, BMI, WHR, waist or hip girth. The magnitude of change after RES training compared to END training was significantly greater in weight (P = 0.02), BMI (P = 0.02) and waist girth (P = 0.046) but not WHR or hip girth.

#### Blood pressure

Group means for BP data in response to RES and END training are shown in Fig 3. There were no significant changes in DBP, SBP or MAP following either RES or END training. The magnitude of change between RES compared to END training was significantly different for DBP (P = 0.01), but not SBP or MAP.

#### Blood glucose and insulin

Group means for blood glucose, insulin, and HOMA-IR in response to RES and END training are shown in Fig 3. There were no significant changes in response to either RES or END training in insulin, glucose, or HOMA-IR or significant differences between the modality changes.

#### Blood lipids

Group means for blood lipid data in response to RES and END training are shown in Fig 4. There were no significant changes in response to either RES or END training in cholesterol, triglycerides, LDL, HDL, or cholesterol/HDL ratio or significant differences between the modality changes.

### Variability in risk factor responses: Responder rate and concordance of response

#### Responder rates

*Anthropometry data*. Individual responses to each exercise intervention are plotted in Fig 2. For weight and BMI, significantly more subjects responded positively (i.e., weight loss) following END (45%) compared to RES (27%) (P = 0.03 for difference between modes). For WHR (P = 0.09 for differences between modes) and waist girth (P = 0.42 for differences between modes), a similar number of subjects responded positively (i.e., reduction) following END (58% and 48%, respectively) compared to RES (43% and 41%, respectively).

*Blood pressure*. Individual responses to each exercise intervention are plotted in Fig 3. For SBP, a similar number of subjects responded positively (i.e., SBP reduction) to RES (41%) and END (54%) (P = 0.14 for differences between modes). For DBP, significantly more subjects responded positively (i.e., DBP reduction) following END (56%) compared to RES (34%) (P = 0.02 for differences between modes).

*Blood glucose and insulin*. Individual responses to each exercise intervention are plotted in Fig 3. For insulin (P = 0.52 for difference between modes), glucose (P = 0.55 for differences between modes) and HOMA-IR (P = 0.92 for differences between modes), a similar number of subjects responded positively (i.e., reduced) to RES (39%, 48% and 49%, respectively) and END (46%, 42% and 50%, respectively).

*Blood lipids*. Individual responses to each exercise intervention are plotted in Fig 4. For cholesterol (P = 0.34 for difference between modes), triglycerides (P = 0.45 for differences between modes) and LDL (P = 0.90 for differences between modes) a similar number of subjects responded positively (i.e., reduced) to RES (40%, 40% and 45%, respectively) and END (50%, 48% and 46%, respectively) training. For HDL, a similar number of subjects responded positively (i.e., increased) to RES (43%) and END (44%) training (P = 0.89 for differences between modes).

#### Concordance between response to different modes of training

**Fig 5:**
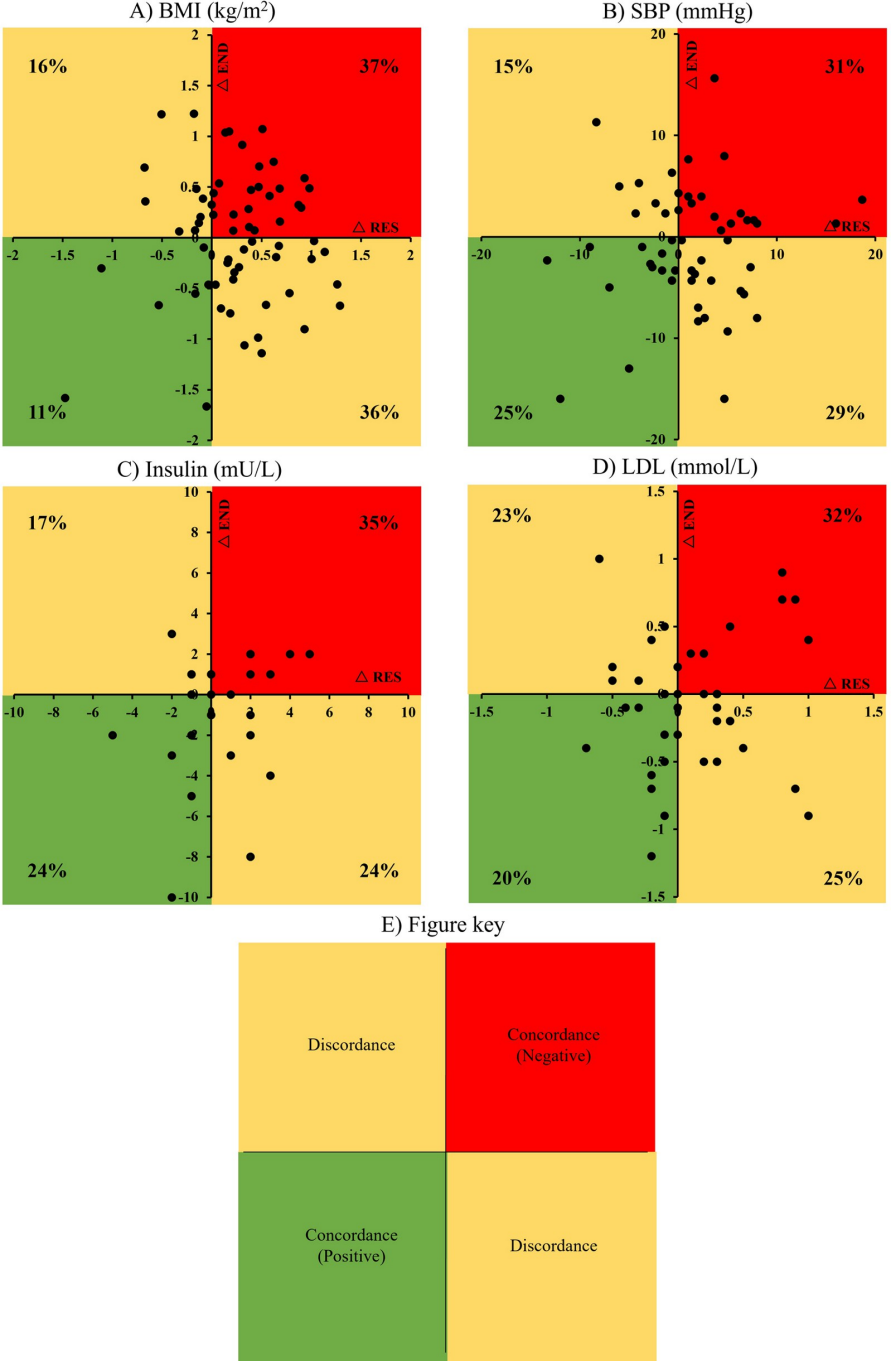
Individual subject exercise intervention change score (Δ) data plotted against one another with response to END on the y-axis and RES on the x-axis. BMI is shown in panel A, SBP is shown in panel B, insulin is shown in panel C, and LDL is shown in panel D. A fig key (E) depicts concordance and discordance for response to RES and END with percentages of responders for each quadrant reported for each variable (A–D).

Concordance results are displayed in Fig 5 and Table 2, which plot each individual’s response for change following both RES and END. Percentages for each quadrant, concordant (both positive or both negative), and discordant, for RES and END interventions, are also presented.

**Table 2:** Concordance between response to different modes of training.

	Positive concordance† %	Negative concordance‡ %	Discordance§ %	Non-responders to RES who responded to END¶ %	Non-responders to END who responded to RES¶ %	P-value (Subjects ‘rescued’ by switching modality (RES vs END))
Weight (kg)	11	37	16+36 = 52	49	30	0.08
BMI (kg/m^2^)	11	37	16+36 = 52	49	30	0.08
WHR	25	25	16+34 = 50	58	39	0.22
Waist girth (cm)	23	31	20+26 = 46	46	39	0.57
SBP (mmHg)	25	31	15+29 = 44	48	33	0.21
DBP (mmHg)	22	29	13+36 = 49	55	31	0.06
Insulin (mU/L)	24	35	17+24 = 41	41	33	0.65
Glucose (mmol/L)	19	28	30+23 = 53	45	52	0.66
HOMA-IR	26	22	26+26 = 52	54	54	0.85
Cholesterol (mmol/L)	16	25	23+36 = 59	59	48	0.42
Triglycerides (mmol/L)	27	36	14+23 = 37	39	28	0.42
LDL (mmol/L)	20	32	23+25 = 48	44	42	0.87
HDL (mmol/L)	11	30	27+32 = 59	47	52	0.78

RES = resistance, END = endurance, BMI = body mass index, WHR = waist to hip ratio, LDL = low-density lipoprotein, HDL = high-density lipoprotein, HOMA-IR = homeostatic model assessment for insulin resistance, SBP = systolic blood pressure, DBP = diastolic blood pressure.

Values should be considered in the context of quadrant plots that graphically depict all individualized subject data (e.g., Fig 4).

†positive concordance means beneficial risk factor response to both forms of training.

‡negative concordance means a detrimental risk factor response to both forms of training.

§discordance means the response to one form of training was beneficial and the other was detrimental (the first number is the number of individuals who had a beneficial response to RES but not END, the second number is the number of individuals who had a positive response to END but not RES and the third number is the sum of these or the total number of individuals who had a beneficial response to at least one form of training).

¶values in these columns were calculated by dividing the number who responded positively after changing modalities by the total number of non-responders for that variable (see text and Fig 4 for details).

*Anthropometry data*. For weight and BMI responses to RES and END, positive concordance (i.e., weight loss to both RES and END) was 11%, negative concordance (weight gain to both modes) was 37% and discordance was 16% and 36% (Fig 5A and Table 2). Non-responders to RES for weight (37+36 = 73% of individuals who gained weight) had a 49% ((36/(36+37))*100) chance of responding with weight loss to END, whereas non-responders to END (16+37 = 53% of individuals gained weight) had a 30% chance ((16/(16+37))*100) of responding with weight loss to RES.

For WHR responses to RES and END, positive concordance was 25%, negative concordance was 25% and discordance was 16% and 34% (Table 2). Non-responders to RES for WHR (25+34 = 59% of individuals) had a 58% ((34/(34+25))*100) chance of responding to END, whereas non-responders to END (16+25 = 41% of individuals) had a 39% ((16/(16+25))*100) chance of responding to RES.

For waist girth responses to RES and END, positive concordance was 23%, negative concordance was 31% and discordance was 20% and 26% (Table 2). Non-responders to RES for waist girth (31+26 = 57% of individuals) had a 46% ((26/(26+31))*100) chance of responding to END, whereas non-responders to END (20+31 = 51% of individuals) had a 39% ((20/(20+31))*100) chance of responding to RES.

*Blood pressure*. For SBP responses to RES and END, positive concordance was 25%, negative concordance was 31% and discordance was 15% and 29% (Fig 5B and Table 2). Non-responders to RES for SBP (60% of individuals) had a 48% chance of responding to END, whereas non-responders to END (46% of individuals) had a 33% chance of responding to RES.

For DBP responses to RES and END, positive concordance was 22%, negative concordance was 29% and discordance was 13% and 36% (Table 2). Non-responders to RES for DBP (65% of individuals) had a 55% chance of responding to END, whereas non-responders to END (42% of individuals) had a 31% chance of responding to RES.

*Blood glucose and insulin*. For insulin responses to RES and END, positive concordance was 24%, negative concordance was 35% and discordance was 17% and 24% (Fig 5C and Table 2). Non-responders to RES for insulin (59% of individuals) had a 41% chance of responding to END, whereas non-responders to END (52% of individuals) had a 33% chance of responding to RES.

For glucose responses to RES and END, positive concordance was 19%, negative concordance was 28% and discordance was 30% and 23% (Table 2). Non-responders to RES for glucose (51% of individuals) had a 45% chance of responding to END, whereas non-responders to END (58% of individuals) had a 52% chance of responding to RES.

For HOMA-IR responses to RES and END, positive concordance was 26%, negative concordance was 22% and discordance was 26% and 26% (Table 2). Non-responders to RES for HOMA-IR (48% of individuals) had a 54% of responding to END, whereas non-responders to END (48% of individuals) had a 54% chance of responding to RES.

*Blood lipids*. For cholesterol responses to RES and END, positive concordance was 16%, negative concordance was 25% and discordance was 23% and 36% (Table 2). Non-responders to RES for cholesterol (61% of individuals) had a 59% chance of responding to END, whereas non-responders to END (48% of individuals) had a 48% chance of responding to RES.

For triglycerides responses to RES and END, positive concordance was 27%, negative concordance was 36% and discordance was 14% and 23% (Table 2). Non-responders to RES for glucose (59% of individuals) had a 39% chance of responding to END, whereas non-responders to END (50% of individuals) had a 28% chance of responding to RES.

For LDL responses to RES and END, positive concordance was 20%, negative concordance was 32% and discordance was 23% and 25% (Fig 5D and Table 2). Non-responders to RES for LDL (57% of individuals) had a 44% chance of responding to END, whereas non-responders to END (55% of individuals) had a 42% chance of responding to RES.

For HDL responses to RES and END, positive concordance was 11%, negative concordance was 30% and discordance was 32% and 27% (Table 2). Non-responders to RES for HDL (57% of individuals) had a 47% chance of responding to END, whereas non-responders to END (62% of individuals) had a 52% chance of responding to RES.

### Comprehensive risk factor response to RES and END interventions

**Fig 6:**
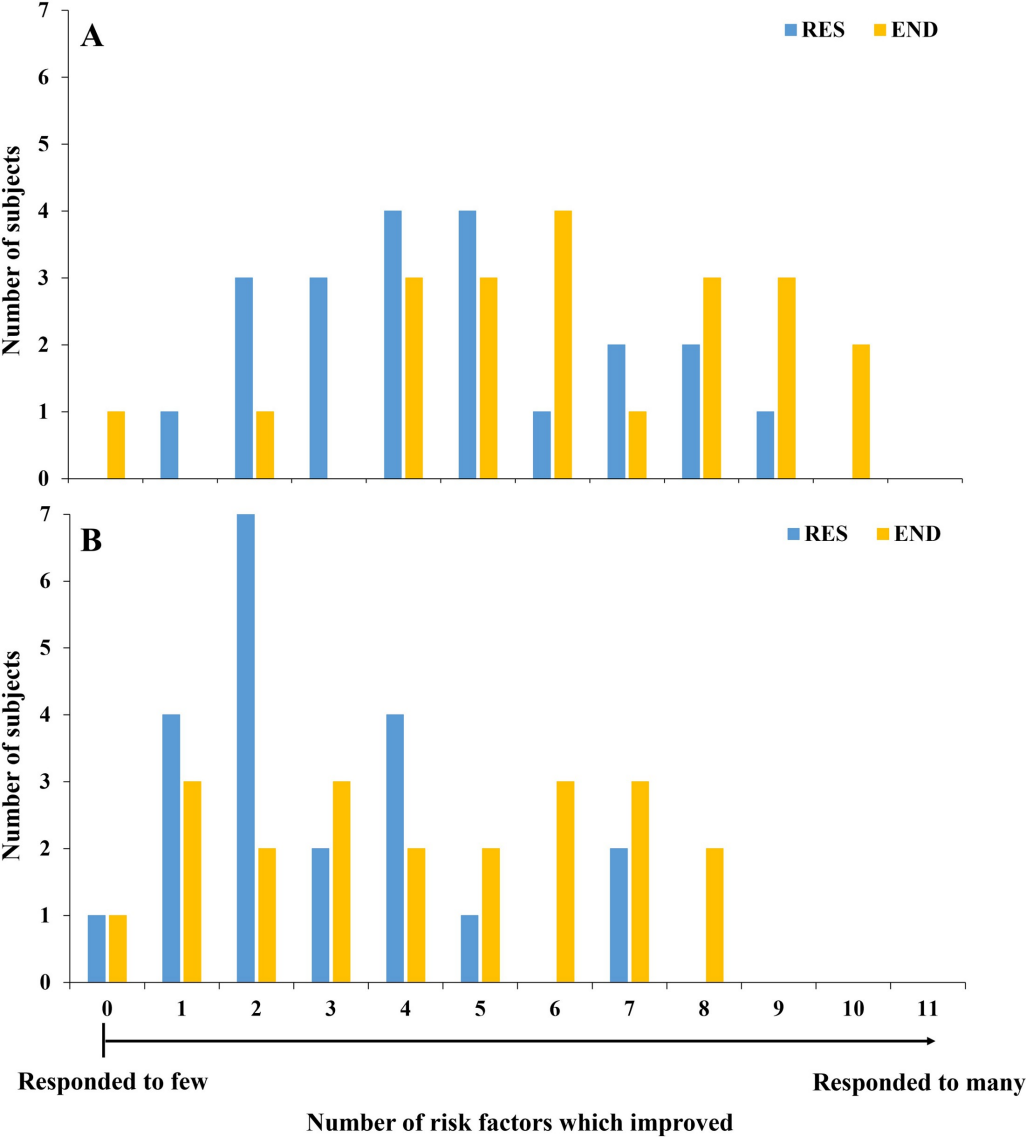
Displays the number of subjects who responded positively to outcome variables. The x axis reflects the number of variables to which individuals responded positively. Only subjects that had data available for every variable at all four time points were included (N = 21). Included variables are cholesterol, triglyceride, LDL, HDL, glucose, HOMA-IR, SBP, DBP, BMI, WHR, waist girth. Panel (A) reflects response defined as any beneficial response in an outcome variable, whereas (B) reflects the number of subjects who responded beneficially with a clinically meaningful change, as indicated by the dotted lines in Figs 2–4.

Twenty-one subjects had universally complete data available for all 11 CV risk factors (cholesterol, triglyceride, LDL, HDL, glucose, HOMA-IR, SBP, DBP, BMI, WHR, waist girth) measured pre and post intervention in response to both RES and END training (Fig 6A). Following RES training, individuals responded beneficially to between one and nine variables. Following END training, one subject did not respond beneficially to any variable, and no subjects responded beneficially to all 11 variables. There was a statistically significant difference (Z = 2.04, P = 0.04) in the median number of risk factors to which subjects improved when they undertook END (median 6 factors) compared to RES (4 factors). Fig 6B presents a similar analysis, where the criterion for benefit reflects clinically meaningful improvement rather than any beneficial change. The median number of risk factors to which subjects improved equal to or beyond the level of clinically meaningful change when they undertook END was 4 factors compared to RES which was 2 factors (Z = 2.21, P = 0.03). The criteria for clinically meaningful change are visually depicted in Figs 2–4 with dotted lines above and below the x axis. The relevant papers used to define clinically meaningful differences are listed in each Fig legend.

### Carry-over and order effects

The 12-wk washout period between training interventions was sufficient for all variables to return to baseline levels. There were no carryover effects present when the two baseline periods were compared (week 0 vs week 24) for any variable. There was no order effect for magnitude of change in any variable.

## Discussion

Despite widespread acceptance as a preventive health intervention, a perception remains that the impact of exercise on CV risk factors can be relatively modest, and pharmacological approaches are often given preference in primary care. This study took advantage of a randomized cross-over design to investigate the within-subject impact on CV risk factors of two typically adopted but physiologically distinct forms of exercise training. Our results in young apparently healthy individuals emphasise that, in the absence of substantive or significant group mean changes in risk factors following either RES or END training, a large proportion of individuals nonetheless respond to these forms of training. In addition, individuals who did not respond to one modality of exercise were typically able to respond to the alternate exercise modality. Finally, individuals who did not respond positively in terms of a specific risk factor, were capable of responding in a beneficial manner when other risk factors were considered. These data suggest that non-responders or low-responders to exercise interventions prescribed at levels currently recommended for health [30, 31] can benefit by changing the exercise modality, emphasising the importance of considering exercise as an intervention that should be personalised for optimal CV health gain within individuals.

To the best of our knowledge, this is the first study to have used a cross-over design to compare the impacts of END and RES on a broad array of CV risk factors, *within* subjects. However, some studies have summarised the impacts of these modalities in *different* groups of subjects, based on changes in group means. A systematic review conducted by Chudyk and Petrella in 2011 [32] included 34 studies between 1970 and 2009 that investigated the effect of END or RES training on clinical markers of CV risk including glycaemic control, dyslipidaemia, blood pressure and body composition in patients with type 2 diabetes. The authors concluded that END exercise alone, or in combination with RES, improved glycaemic control, SBP, triglycerides and waist circumference, whereas RES training alone did not have any significant effect on CV markers [32]. Similarly, a study by Suleen Ho *et al*., in 2012 [15] investigated the effect of 12 weeks endurance (n = 15), resistance (n = 16) or combined (n = 17) exercise interventions, compared to control (n = 16), on CV risk factors in overweight or obese individuals. It was concluded that resistance or combined exercise interventions resulted in improvements in CV risk profiles compared to no exercise. In the current study there were no significant group mean changes in any of the CV risk factors measured, with the exception of weight and BMI. Our recent publication describing imaging-based body composition (DXA) changes showed that there was a significant *increase* in lean body mass in response to END and RES, resulting in an increase in overall body weight, whilst fat mass decreased [16]. The fact that other CV risk factors failed to significantly improve may have been because the participants were young and relatively healthy compared to previous studies in clinical and older populations. As stated above, it is important to emphasise that, despite the absence of mean group changes in our subjects, consideration of individualised data indicates that a large proportion of subjects responded to training at levels that may be considered clinically meaningful. For example, 27% (RES) and 42% (END) of subjects demonstrated beneficial SBP changes ≥2 mmHg [23], 40% (RES) and 45% (END) of subjects demonstrated beneficial HOMA-IR changes ≥0.20 units [24], 28% (RES) and 36% (END) of subjects demonstrated beneficial LDL changes ≥0.102mmol/L [25] and 43% (RES) and 44% (END) of subjects demonstrated beneficial HDL changes ≥0.100 mmol/L [25].

In the past decade, the variability in human responsiveness to exercise training has gained attention. This was initially prompted by an analysis of non-response to exercise training by Bouchard *et al*., [9] who reviewed a number of intervention studies [33–39] totalling 1,687 men and women that included “adverse” metabolic responders to regular exercise in terms of CV risk factors. Prevalence of adverse responders for SBP was 12.2%, HDL-cholesterol 13.3%, triglycerides 10.4% and fasting insulin was 8.4%, with 7% of participants experiencing adverse response to two or more risk factors [9]. Our findings in the present study, which involved centre-based and supervised exercise training undertaken at identical exercise intensities by all subjects, generally reinforce the findings of Bouchard *et al*., in that we observed a substantial proportion of subjects (51–73% for RES and 42–58% for END) who did not respond positively to training. These findings suggest that low response to guideline-based exercise in CV risk factors can occur, and that identifying ways to prevent low responses provides a compelling foundation for personalised exercise prescription [9].

One approach to achieving better exercise outcomes is to modify the prescription. While some studies have focused on exercise intensity and frequency in this regard [40, 41], not all individuals are capable of safely performing exercise at ever more intense levels. We therefore investigated whether changing the *modality* of exercise could enhance the response to training and stimulate positive physiological and health adaptations [42]. Some previous cross-over designed studies have investigated changes in fitness (VO_2_max) in response to END and RES training. Hautala *et al*., [43] reported that individuals who failed to improve their VO_2_max in response to END training were able to improve their VO_2_max in response to RES training. Our data from the subjects in the current experiment for VO_2_max [17] and body composition [16] support this finding, indicating that individual patterns of non-response may vary by training mode. For the CV risk factor outcomes reported in the current paper, we also observed that 39–59% (range across all variables) of individuals who did not respond to RES, changed their response to training by switching to END. Similarly, 28–54% (range across all variables) of individuals who did not respond to END, changed their response by switching to RES. We therefore conclude that modification of exercise modality in subjects who initially appear to respond modestly, can provide a powerful approach to optimising the benefits of training.

Another perspective on the low responder phenomenon is that exercise is a systemic stimulus that affects multiple physiological pathways, and that different individuals will express the benefits of exercise in distinct ways. Where one individual may improve BP and body weight following exercise, another may experience improvements in their lipid profile. When we assessed responses across 11 different risk factors in a sub-group of 21 subjects who had complete data for all measures in response to both training modalities, it was apparent that while most subjects responded positively in terms of some risk factors, relatively few responded positively to all. There was a significantly higher median number of risk factors that showed beneficial change in response to END (6 factors), than for RES (4 factors). A similar pattern was apparent when change was defined as equal to or beyond the level of clinical significance, where the number of risk factors that showed a beneficial change in response to END was 4 factors and RES was 2 factors (Fig 6B). Ultimately, where exercise prescription targeting specific risk factor modification (e.g., BP, or blood lipids) is the goal, it may be beneficial to change exercise modality if the initial approach is relatively ineffective. Our results indicate that, should lack of response in a particular risk factor be apparent, it remains likely that other factors are beneficially modified.

This study presents changes in traditional CV risk factors in response to two commonly utilised and ecologically valid modes of exercise training. It is important to reinforce, in accordance with previous evidence [1, 6], that exercise has beneficial impacts beyond those apparent in traditional CV risk factors. Several papers which have aimed to define the mechanisms that contribute to the substantial CV benefit of exercise, have typically concluded that changes in traditional risk factors contribute <50% to the clinical benefit [44, 45]. Other mechanisms, such as novel risk factor and direct biomechanical impacts on vascular function and health, for example through change in endothelial function [1, 6, 46], may fill this risk factor gap.

This study has strengths, but also limitations. The lack of a non-exercise control group could be considered a limitation, but the effects of exercise on CV risk factors are well documented, justifying the superiority design comparing RES to END. This study included a large number of outcome measures collected over multiple laboratory attendances, hence there was some variation in the number of data points across different outcome measures. For example, the N for most of the blood sample variables was lower than that of the anthropometry or BP variables. Reasons for this included some subjects choosing not to have a blood sample, difficulty in venous access, and haemolysis of samples. Repeating this study in a higher risk or aged population may yield different results than this study of apparently healthy and relatively young subjects. Another limitation relates to our control over variables such as diet, which may impact on levels of trainability and the proportion of responders/non responders. Finally, this study did not investigate the potential benefit of combining END and RES training in an intervention, although we have successfully applied such an approach in the past [47, 48]. A recent study by Schroeder *et al*., [49] investigated the effect of 8 weeks of RES training (n = 17), END training (n = 17) and a combined training intervention (n = 18) versus a control group (n = 17) on CV risk factors. The authors reported that combined training, but neither END or RES alone, significantly reduced BP and concluded that among individuals at an increased risk of CV disease, combined training may provide comprehensive benefits compared to RES or END alone [49]. Individual variability in responsiveness was not addressed in this study. Future within-subjects studies comparing a combined training approach to RES and END alone would be ideal, if somewhat difficult to design utilising a cross-over design.

The findings presented in this study help to answer some fundamental questions surrounding ways to modify CV risk factors with exercise training and may assist in informing clinicians and exercise professionals regarding exercise prescription. This study reported that, on an average group level, 12 weeks of RES or END did not change most risk factors in apparently healthy individuals. However, a large proportion of subjects nonetheless showed robust positive responses, whilst others responded modestly or negatively. Exercise modality had a significant impact on the proportion of subjects who responded, and concordance data indicated that non-response to one mode of training does not imply non-response to other training modes. Finally, although the effect of an exercise intervention on a single risk factor may be modest, the effect of exercise on overall CV risk profile can be dramatic [50]. Exercise modality is an important consideration when planning and executing exercise prescriptions targeted at risk factor modification in individuals.

## Supporting information

S1 Checklist CONSORT checklist. (DOC)

S1 File Ethics committee trial protocol. (DOCX)

S1 Data Raw data. (XLSX)

## Abbreviations

CV: cardiovascular
BP: blood pressure
SBP: systolic blood pressure
DBP: diastolic blood pressure
HR: heart rate
RES: resistance training
END: endurance training
BMI: body mass index
WHR: waist to hip ratio
LDL: low-density lipoprotein
HDL: high-density lipoprotein
HOMA-IR: homeostatic model assessment for insulin resistance
1RM: one-repetition max
VO_2_max: maximal exercise test
