# Supplementary material for: Endurance versus resistance training in treatment of cardiovascular risk factors: A randomized cross-over trial
Source: PLoS One. 2022 Sep 6;17(9):e0274082. doi: 10.1371/journal.pone.0274082 (PMC9447867; doi:10.1371/journal.pone.0274082)
Supplement: S1 File — (DOCX) [file pone.0274082.s002.docx]

|  | | **Human Research Ethics Office** Research Services, MBDP: M459  The University of Western Australia 35 Stirling Highway Crawley WA 6009  Phone: +61 8 6488 4703  Fax: +61 8 6488 8775  Email: [hreo-research@uwa.edu.au](mailto:hreo-research@uwa.edu.au)  research.uwa.edu.au/staff/human-research |
| --- | --- | --- |
| **Application for Human Research Ethics Review**  (Please use the latest version of this [form](http://www.research.uwa.edu.au/staff/human-research/human-ethics) every time you submit an application)  Use this form to apply for ethical review of research involving people to be carried out at The University of Western Australia. Ethical review of research is a necessary pre-requisite for research involving. This includes even research that studies data about people and observation of people.  Submit completed form to Human Research Ethics Office: [hreo-research@uwa.edu.au](mailto:hreo-research@uwa.edu.au)  Email or phone the office for assistance with this form – contact details are in the letterhead above. | | |
| **Do you seek Exemption from Review?** | **If Yes, stop here**. Please use the form **Application for Exemption from Ethics Review**, available at the [Human Research Ethics website](http://www.research.uwa.edu.au/staff/human-research/approvals/exemption) ([click here](http://www.research.uwa.edu.au/staff/human-research/approvals/exemption)). | |

| **Have you received ethics approval from another Ethics Review Committee?** | No |
| --- | --- |

**Continue below if you seek UWA review of your human research ethics project.**

| 1. **Project title:** | Towards exercise as personalised medicine: Is non-response to exercise generic, genetic or modality dependent? |
| --- | --- |

| 1. **Chief Investigator, or Supervisor in case of student research:** | | | | | |
| --- | --- | --- | --- | --- | --- |
| **Name** | W/Prof Daniel Green | | | | |
| **School, Centre or Faculty** | School of Sport Science, Exercise and Health | | | **StaffId #:** | 00016392 |
| **Telephone** | 6488 5609 | **Email:** | Danny.green@uwa.edu.au | | |

| **Additional researchers / co-investigators, including students and non-UWA investigators** | | | | |
| --- | --- | --- | --- | --- |
| **Researcher** | **Title, given name, and family name** | **School, centre, institution** | **Staff # or student #** | **Co-inv, student,** |
| **2** | Dr Louise Naylor | School of Sport Science, Exercise and Health | 00050763 | UWA Staff |
| **3** | W/Prof Nick de Klerk | Telethon Kids Institute | Click here to enter text. | UWA Staff |
| **4** | Dr Lawrence Dembo | Envision Imaging | Click here to enter text. | Non-UWA staff |
| **5** | W/Prof David Mackey | Centre for Ophthalmology and Visual Science | Click here to enter text. | UWA Staff |

[Insert additional rows for additional investigators]

| Hyperlinked cross references to the *National Statement on Ethical Conduct in Human Research* are abbreviated below as NS §section.chapter. If you have any doubt about the meaning or purpose of a question, please use those hyperlinked references to read the definitive explanation. |
| --- |

| 1. **Team Expertise** | W/Prof Green is an expert in cardiovascular physiology, and head of the cardiovascular research group at the School of Sports Science, Exercise and Health, which is fully equipped to carry out the assessments to be utilised in this study.  Dr Naylor is an accredited exercise physiologist and researcher at the University with extensive experience undertaking all of the vascular and exercise testing protocols proposed in the present study.  W/Prof DeKlerk has over 30 years experience as a biostatistician with epidemiology studies, and has published and presented widely. Prof De Klerk is also the head of the WA twin registry (WATR). Dr Dembo has a Fellowship in Cardiac Imaging, and is accredited by the conjoint committee of the CSANZ/RANZCR at the highest level. He has a Level 3 MRI equivalency by the Society of Cardiovascular Magnetic Resonance accreditation guidelines. He maintains his clinical Cardiology and will provide expertise in the collection, analysis and interpretation of the cardiac data.  W/Prof David Mackey’s research focus in in hereditary eye diseases, particularly the clinical and molecular genetic aspects of the disease. He will provide the expertise required to conduct and analyse the eye scans.  [NS §3.3.5](http://www.nhmrc.gov.au/book/chapter-3-3-interventions-and-therapies-including-clinical-and-non-clinical-trials-and)*, Explain how the research team has sufficient skills and experience to conduct the proposed clinical research.* |
| --- | --- |

| 1. **Expected project start and end?** | Start date: | 1-Sep-14 | End date: | 31-Dec-19 |
| --- | --- | --- | --- | --- |

| 1. **How is the research funded?** | Funding for this project has been sought from the Australian Research Council (Pending).  Please provide project application title for funding if different from this application.  Funder, Scheme, reference number (as applicable). Please provide UWA grant reference number if applicable: RA/1/####/### Is funding sought that is not yet approved? Respond to the potential conflict of interest question at the end of this form. |
| --- | --- |

| 1. **Aims of this project.**   **(Layman’s terms)** | The aim of this project is to investigate the phenomenon of response/non-response to exercise training in individuals and to determine whether this pattern is different if distinct forms of exercise are adopted. It will also address whether genetics play a role in the response profile. This study is highly relevant because the benefits of exercise might be optimised by individualising exercise prescription.  Describe the purpose of this research *in layman’s terms please*. About 300 words recommended.  **Attach the research proposal to supply additional detail, background, research design, analysis and procedures** where relevant. |
| --- | --- |

| 1. **Research design (include analysis & procedures if any)** | Include information about procedures to be carried out and your analytic methodology where appropriate.  Explain if different groups of participants will be treated differently.  Subjects will be randomised to undertake a 3-month program of either resistance or endurance exercise training, according to computer-generated randomisation. After 3 months of training and a 3-month wash out period, each subject will crossover to start a similar period (3 months) of training with the alternate intervention modality.  **Exercise intervention modalities**  The exercise training programs will be similar to those used in our previous published experiments (100+ papers) and consist of individually-tailored exercise designed and progressed according to accepted exercise science principles. Subjects will attend three supervised training sessions per week at the research gymnasium at the University of WA for the duration of the intervention.  ***Aerobic Exercise Training Program***  Training programs will be individually tailored to each person using their VO_2peak_ and will start at a moderate to light intensity. Each training session will involve 20 minutes of cycling and 20 minutes of treadmill walking/running, separated by a 6.5 min rest period.  ***Resistance Exercise Training Program***  Participants will be shown how to safely use all weight machines, and then under supervision will perform 15 to 20 repetitions in 60 sec followed by a 30 sec changeover period. A variety of exercises will be chosen to target specific muscle groups.  ***Outcome measures:***   1. ***Blood tests:*** A small sample of blood (~20mL, equivalent to 1 tablespoon of blood) will be collected via a venipuncture to measure enzyme markers and traditional cardiovascular risk factors such as insulin, glucose, lipids and some markers of platelet function. 2. ***Vascular function and structure:*** Duplex ultrasonography will be used to assess conduit artery structure and function (wall thickness, hyperemic response to ischemic exercise*;* an index of arterial lumen remodelling, flow-mediated dilation; FMD and sublingual glyceryl trinitrate administration; GTN). Measurements will be acquired in the carotid, brachial and superficial femoral arteries. Our laboratory group has extensive experience in all of these measures, which will be conducted in accordance with the recent guidelines which our group was invited to prepare. We will also assess the health of the small blood vessels in the skin of the forearm (using laser Doppler and a localised painless heating protocol) and eyes (retinal camera). These outcomes are directly relevant to cardiovascular health. 3. ***Cardiac outcome measures:*** Echocardiographic images will be acquired using a 1.5- to 4-MHz phased array transducer on a commercially available ultrasound system (Vivid I, GE Medical, Horton, Norway). Standard B-mode and M-mode measures include LVIDd, LVIDs, IVST, PWT LVmass, and LV volumes will be calculated. Novel measurement of LV mechanics, such as stress and strain, will be assessed post-hoc using EchoPAC software. Cardiac Magnetic Resonance imaging will also be used*.* Subjects will be scanned pre and post training using a 1.5-T cardiac CMR scanner (Siemens Magnetom Espree, Erlangen, Germany). The cardiac CMR analysis is performed with specialized software (ARGUS, Siemens) by an observer who was blinded to subject, group allocation and scan time-point. We recently used this approach in our published work on cardiac changes to 6 month training programs. 4. ***Cerebrovascular outcome measures:*** *Intracranial blood velocity assessment using transcranial* *Doppler-* Cerebral blood flow (CBF) is assessed by combining bilateral measures of anterior (ACA), middle (MCA) and posterior cerebral artery (PCA) flow velocities using state-of-the-art transcranial Doppler (TCD) techniques. We will also assess CBF during a series of standard, physiological tests, including: cerebrovascular CO2 reactivity, dynamic cerebral autoregulation, neurovascular coupling and cerebral artery blood flow responses to exercise. 5. ***Other outcome measures:*** At entry, midpoint and the end of the intervention period (3 months), cardiorespiratory fitness will be assessed via VO2peak testing on a treadmill, muscular strength using the 1RM approach and blood pressure using ambulatory 24hr measures and body composition using dual emission x-ray absorptiometry (DXA). |
| --- | --- |

| 1. **Sampling** | We are seeking 50 pairs each of mono- and di-zygotic (these being same sex) twins  Living in the Perth metropolitan area, Western Australia  **List any other relevant criteria.**  Aged between 10 and 40 yrs  No previous diagnosis of cardiovascular or metabolic disease (i.e. diabetes, heart or blood vessel disease)  Free from genetic disorders or significant organ system illness or musculoskeletal disorders that would prevent full participation in an exercise training program  Relatively inactive lifestyle (<2 x 30-min moderate exercise/wk in previous 6 months)  To be available for the full 12 month duration of the study  If using a sample, explain how the size and profile of the sample to be recruited is adequate to answer the research question. |
| --- | --- |

| 1. **Recruitment methods:** | We will recruit pairs of twins via advertisement and through the Australian Twin registry (ATR). We have secured approval from the ATR to undertake this study using their database.  In addition, participants will be recruited from the local community in surrounding suburbs via advertisement (e.g. community newspaper media release).   Potential participants will be invited to a screening interview (via telephone) to determine eligibility for study participation. At this interview, information regarding lifestyle behaviours and medical history will be obtained.  Please attach copies of advertisements, flyers, posters, emails, etc. |
| --- | --- |

| 1. **Data management and publication plan:** | Data will be de-identified, coded, backed-up and securely stored on servers which are password and algorithm protected. All data will be analysed by blinded observers and coded for retrieval. Paperwork will be stored in a locked filing cabinet in a secure building on the UWA campus and, as per our standard practice, will be retained for 10 years following publication or project completion. The Australian twin registry database will be utilised for recruitment purposes and all existing protocols for contact with subjects will be observed.  Communication of results. This work is human-based and directly translatable and it therefore often features in newspapers and on websites following presentations at national and international conferences. Scientific aspects of the results will be publicised through peer reviewed publications and presentations at conferences.  Research records must be retained for a minimum of 7 years after date of publication or project completion, whichever is later ([Western Australian University Sector Disposal Authority](http://intranet.uwa.edu.au/archives/__data/page/38711/Western_Australian_University_Sector_Disposal_Authority_SD2011011.PDF)).  How and where will you record, store, share, transmit, and archive your data? Discuss retention, security and data sharing plans. How will you publish / disseminate your work.  Consult the [*Australian code for the responsible conduct of research*](http://www.nhmrc.gov.au/_files_nhmrc/publications/attachments/r39.pdf), section 2; and, the [UWA Research Management Toolkit](http://www.is.uwa.edu.au/research/research-data-management-toolkit). |
| --- | --- |

**Ethics Themes: risk and benefit, consent**

| 1. **Potential harms or risks to participants?**   [NS §2.1](http://www.nhmrc.gov.au/book/chapter-2-1-risk-and-benefit) | A 400 microgm dose of glyceryl trinitrate (GTN) will be administered sublingually. Side effects (if any) are usually mild and transient and can include small increases or decreases in heart rate, headache and/or dizziness or weakness when standing suddenly, with less common side effects including nausea. These resolve rapidly and spontaneously, although any persistent headache can be managed using paracetamol. Participants who experience any side effects will be monitored in the laboratory until their symptoms resolve, and/or their blood pressure (both lying and standing) normalise. We have extensive published experience using this drug dose and have numerous approvals from this committee. WE have generic approval for the use of GTN is physiological studies such as this from the UWA committee (RA/4/1/6158)  Some participants may experience mild discomfort associated with the cuff inflation on the arm used during the FMD protocol. However, this subsides immediately when the cuff is deflated and there are no long-term side effects associated with this procedure.  Similarly, during the routine blood pressure and ambulatory blood pressure assessments, participants may feel discomfort associated with cuff inflation. However, this sensation is transient.  Generally, pain and discomfort associated with venepuncture is very minor, usually no more than what is experienced during a routine blood test. Some individuals may experience a mild sting when the needle penetrates the skin. Few will develop minor bruising at the site of collection. Some participants may experience minor emotional stress as a result of the blood testing procedure, however efforts will be made to ensure minimise this effect, such as ensuring an experienced trained phlebotomist collects samples with participant in the supine position. Should there be any adverse effects as a result of the blood test (e.g. participant experiencing a vasovagal episode), a second researcher will be on call to provide support.  During the aerobic fitness test, participants will be encouraged to perform at their best, however as this is a volitional test and can be terminated any time. Participants will be informed of this prior to commencing the tests.  The training sessions may also cause some acute discomfort, as sub-maximal exercise efforts may be required. However each participant will be extensively monitored by an exercise physiologist, throughout the sessions and activity altered accordingly. Participants may also experience some delayed onset muscle soreness (DOMS) as a result of the training sessions, although these symptoms should resolve after 24-48 hours. Throughout the testing and training procedures, participants will be closely monitored using heart rate monitors and subjective exertion scales (e.g. Borg rating of perceived exertion). In addition, careful screening procedures will employed prior to participants commencing any testing/training. All personnel involved will be trained in CPR and extensive First Aid and defibrillator facilities will be made available in the laboratory and training settings.  See [NS §2.1](http://www.nhmrc.gov.au/book/chapter-2-1-risk-and-benefit) - consider illness or injury, potential side effects; but also include potential embarrassment, economic loss, exposure to prosecution, anything stressful, noxious or unpleasant. Ensure you address these in your Participant Information Forms (PIF) if you are using those.  **Explain how this research justifies the burden and risks to participants?** |
| --- | --- |

| 1. **Potential harms or risks to researchers?** | Nil  Does this work open the research team to direct or indirect risk? Please explain. |
| --- | --- |

| 1. **Will participants be given financial or non-financial incentives?** | | Yes:  No:   **🡻** | [NS §2.2.10-11](http://www.nhmrc.gov.au/book/chapter-2-2-general-requirements-consent) concerns inducements. |
| --- | --- | --- | --- |
| **Please describe:** | Click here to enter text.  NS §2.2.10 - payment that is disproportionate to the time involved, or any other inducement that is likely to encourage participants to take risks, is ethically unacceptable. | |  |

| 1. **Will all participants provide consent?** | | Yes:  No:  **🡻** | [NS §2.2](http://www.nhmrc.gov.au/book/chapter-2-2-general-requirements-consent) concerns issues of consent. [NS §2.3](http://www.nhmrc.gov.au/book/chapter-2-3-qualifying-or-waiving-conditions-consent) concerns potential waiver of consent.  *Attach Participant Information and Consent Forms*. | |
| --- | --- | --- | --- | --- |
| **How will you obtain consent, or justify a waiver?** | Written informed consent will be obtained from all participants, and in the case of those under the age of 18 years, written consent will be obtained from their parent/guardian as well (specific PIF attached).  Describe how you will deal with [§2.2](http://www.nhmrc.gov.au/book/chapter-2-2-general-requirements-consent) and [§2.3](http://www.nhmrc.gov.au/book/chapter-2-3-qualifying-or-waiving-conditions-consent) of the *National Statement* regarding consent. If you request waiver of consent you must address NS [§2.3](http://www.nhmrc.gov.au/book/chapter-2-3-qualifying-or-waiving-conditions-consent). For a waiver, please also see the following section regarding the *Privacy Act*. | | |  |
| **Privacy Act 1998, Sections §95 and §95A** | If you do not have written consent AND you are requesting a waiver of consent to access Commonwealth or private-sector data, you will need to justify how the public interest value of your research relevant to public health or public safety out-weigh the public interest in the protection of privacy. There are guidelines to assist you with this and you need to use them to make your case to the HREC. To use health information from a Commonwealth Government agency, see: Guidelines Under Section 95 of the Privacy Act 1988 available at: <http://www.nhmrc.gov.au/_files_nhmrc/publications/attachments/e26.pdf> To use health information from a private sector source, see: Guidelines approved under Section 95A of the Privacy Act 1988 available at: <http://www.nhmrc.gov.au/_files_nhmrc/publications/attachments/e43.pdf> | | |  |
|  | **Do you need health data from Commonwealth agencies (§95)?** | | Yes |  |
|  | **Do you need health data from private-sector sources (§95A)?** | | Yes |  |

| 1. **Will the research use deception, concealment or incomplete disclosure?** | | Yes:  No:   **🡻** | [NS §2.3](http://www.nhmrc.gov.au/book/chapter-2-3-qualifying-or-waiving-conditions-consent) discusses use of deception, covert observation, concealment, and incomplete disclosure. |
| --- | --- | --- | --- |
| **Please describe:** | Click here to enter text.  Explain why this is essential to the research aims. How will participants be de-briefed after the experiment? | |  |

**Ethical considerations specific to research methods or fields**

| 1. **Will you make video, photograph, or audio recordings?** | | Yes:  No:   **🡻** | [NS §3.1](http://www.nhmrc.gov.au/book/chapter-3-1-qualitative-methods) provides guidance on recording. |
| --- | --- | --- | --- |
| **Please describe:** | The ultrasound images used for the vascular will be recorded as avi images and kept for off line analysis. All files will be de-identified and kept on a password protected computer  Address cultural issues if applicable. Ensure you explain storage of this material in your data management plan (above). Ensure you advise of this in your Participant Information and Consent forms. | |  |

| 1. **Use of Qualitative Methods** | | Yes:  No:   **🡻** | See special considerations of qualitative methods in [NS §3.1](http://www.nhmrc.gov.au/book/chapter-3-1-qualitative-methods) |
| --- | --- | --- | --- |
| **Comments regarding** [NS §3.1](http://www.nhmrc.gov.au/book/chapter-3-1-qualitative-methods) | Click here to enter text. | |  |

| 1. **Use of data from data banks** | | Yes:  No:   **🡻** | See special considerations of data banks in [NS §3.2](http://www.nhmrc.gov.au/book/chapter-3-2-databanks) |
| --- | --- | --- | --- |
| **Comments regarding** [NS §3.2](http://www.nhmrc.gov.au/book/chapter-3-2-databanks) | Click here to enter text. | |  |

| 1. **Interventions, therapies, trials** | | Yes:  No:   **🡻** | See special considerations of interventions, therapies, clinical and non-clinical trials in [NS §3.3](http://www.nhmrc.gov.au/book/chapter-3-3-interventions-and-therapies-including-clinical-and-non-clinical-trials-and) |
| --- | --- | --- | --- |
| **Comments regarding** [NS §3.3](http://www.nhmrc.gov.au/book/chapter-3-3-interventions-and-therapies-including-clinical-and-non-clinical-trials-and) | All participants will receive two exercise training programs, including aerobic-type exercises like stationary cycling, treadmill walking/jogging, and resistance exercises using hand held and machine weights.  The programs will be supervised and similar to those used in our previous published experiments (100+ papers) and consist of individually-tailored exercise designed and progressed according to accepted exercise science principles by an accredited exercise physiologist (AEP). Subjects will attend three supervised training sessions per week at the research gymnasium at the University of WA for the duration of the intervention. | |  |

| 1. **Human Tissue** | | Yes:  No:   **🡻** | See special considerations of tissue use in [NS §3.4](http://www.nhmrc.gov.au/book/chapter-3-4-human-tissue-samples) |
| --- | --- | --- | --- |
| **Comments regarding** [NS §3.4](http://www.nhmrc.gov.au/book/chapter-3-4-human-tissue-samples) | Click here to enter text.  This includes collection of blood, tissue, bone, fluids, hair, teeth, DNA.  Include explanation of biobank use, and if samples are an ongoing part of the research, or merely to assess entry criteria. | |  |

| 1. **Human Genetics** | | Yes:  No:   **🡻** | See special considerations of genetics in [NS §3.5](http://www.nhmrc.gov.au/book/chapter-3-5-human-genetics) |
| --- | --- | --- | --- |
| **Comments regarding** [NS §3.5](http://www.nhmrc.gov.au/book/chapter-3-5-human-genetics) |  | |  |

**Specific details of interventions, therapies and trials**

| 1. **Are drugs, biological agents or therapeutic devices to be used?** | | Yes:  No:  **🡻** | See the guidelines in [NS §3.3](http://www.nhmrc.gov.au/book/chapter-3-3-interventions-and-therapies-including-clinical-and-non-clinical-trials-and)  **Note:** A Clinical Trials Notification (CTN) or Clinical Trial Exemption (CTX) from [Therapeutic Goods Administration](http://www.tga.gov.au/industry/clinical-trials.htm) (TGA) will be required if this is a clinical trial. |
| --- | --- | --- | --- |
| **Attach full protocol** | A 400 microgm dose of glyceryl trinitrate (GTN) will be administered sublingually as part of the standard protocol used to assess vascular structure and function. | |  |

| 1. **Will invasive procedures be used?** | | Yes:  No:  **🡻** | [NS §3.3](http://www.nhmrc.gov.au/book/chapter-3-3-interventions-and-therapies-including-clinical-and-non-clinical-trials-and) |
| --- | --- | --- | --- |
| **Attach full protocol** | A routine venous puncture will be used to collect a sample of blood for analysis of lipids and metabolic outcomes. The blood collection will be outsourced to a commercial pathology collection centre, as will the analysis of the blood samples. | |  |

| 1. **Will there be a placebo or non-treatment group?** | | Yes:  No:   **🡻** | [NS §3.3](http://www.nhmrc.gov.au/book/chapter-3-3-interventions-and-therapies-including-clinical-and-non-clinical-trials-and) See 3.3.10 in particular. |
| --- | --- | --- | --- |
| **Please describe:** |  | |  |

| 1. **Will (ionising) radiation be used?** | | Yes:  No:  **🡻** | [Review the ARPANZA guidelines](http://www.arpansa.gov.au).  If Yes, you will also need to obtain approval from UWA Health and Safety for this work. |
| --- | --- | --- | --- |
| **Please describe:** | A DEXA scan will be used to assess bone density and body composition. Dr Naylor is licensed and qualified to use the DEXA scanner. Please refer to attached letter from the UWA radiation safety officer. | |  |

**Does the research focus on any of the following groups of people?**

| 1. **Pregnant women, or Human ovum, embryo, or foetus** | | Yes:  No:   **🡻** | See special considerations for work with pregnant women, ova, embryo or foetus in [NS §4.1](http://www.nhmrc.gov.au/book/chapter-4-1-women-who-are-pregnant-and-human-foetus) |
| --- | --- | --- | --- |
| **Comments regarding** [NS §4.1](http://www.nhmrc.gov.au/book/chapter-4-1-women-who-are-pregnant-and-human-foetus) | Click here to enter text. | |  |

| 1. **Children or young people (**< 18 y.o.**)** | | Yes:  No:   **🡻** | See special considerations for work with young people in [NS §4.2](http://www.nhmrc.gov.au/book/chapter-4-2-children-and-young-people) |
| --- | --- | --- | --- |
| **Comments regarding** [NS §4.2](http://www.nhmrc.gov.au/book/chapter-4-2-children-and-young-people) | Written consent will be obtained from parent/guardian of any participant under the age of 18 years. | |  |

| 1. **Dependent or unequal relationships** | | Yes:  No:   **🡻** | See special considerations for work with people in dependent relationships in [NS §4.3](http://www.nhmrc.gov.au/book/chapter-4-3-people-dependent-or-unequal-relationships) |
| --- | --- | --- | --- |
| **Comments regarding** [NS §4.3](http://www.nhmrc.gov.au/book/chapter-4-3-people-dependent-or-unequal-relationships) | Click here to enter text. | |  |

| 1. **Highly dependent on medical care** | | Yes:  No:   **🡻** | See special considerations for work people dependent on medical care in [NS §4.4](http://www.nhmrc.gov.au/book/chapter-4-4-people-highly-dependent-medical-care-who-may-be-unable-give-consent) |
| --- | --- | --- | --- |
| **Comments regarding** [NS §4.4](http://www.nhmrc.gov.au/book/chapter-4-4-people-highly-dependent-medical-care-who-may-be-unable-give-consent) | Click here to enter text. | |  |

| 1. **Cognitive impairment, intellectual disability, or mental illness** | | Yes:  No:   **🡻** | See special considerations for work with people with cognitive impairment in [NS §4.5](http://www.nhmrc.gov.au/book/chapter-4-5-people-cognitive-impairment-intellectual-disability-or-mental-illness) |
| --- | --- | --- | --- |
| **Comments regarding** [NS §4.5](http://www.nhmrc.gov.au/book/chapter-4-5-people-cognitive-impairment-intellectual-disability-or-mental-illness) | Click here to enter text. | |  |

| 1. **Potential exposure of illegal activities** | | Yes:  No:   **🡻** | See special considerations for work with potential exposure of illegal activities in [NS §4.6](http://www.nhmrc.gov.au/book/chapter-4-6-people-who-may-be-involved-illegal-activities) |
| --- | --- | --- | --- |
| **Comments regarding** [NS §4.6](http://www.nhmrc.gov.au/book/chapter-4-6-people-who-may-be-involved-illegal-activities) | Click here to enter text. | |  |

| 1. **Aboriginal or Torres Strait Islander People** | | Yes:  No:   **🡻** | See special considerations for work with indigenous people in [NS §4.7](http://www.nhmrc.gov.au/book/chapter-4-7-aboriginal-and-torres-strait-islander-peoples) |
| --- | --- | --- | --- |
| **Comments regarding** [NS §4.7](http://www.nhmrc.gov.au/book/chapter-4-7-aboriginal-and-torres-strait-islander-peoples) | Click here to enter text. | |  |

| 1. **Are any participants outside Australia?** | | Yes:  No:   **🡻** | [NS §4.8](http://www.nhmrc.gov.au/book/chapter-4-8-people-other-countries) explains specifics of working outside Australia. |
| --- | --- | --- | --- |
| **Countries and approvals:** | Click here to enter text. | |  |

| 1. **Is there a potential conflict of interest?** | | Yes:  No:  **🡻** | [NS §5.4](http://www.nhmrc.gov.au/book/chapter-5-4-conflicts-interest) addresses conflicts of interest in detail. |
| --- | --- | --- | --- |
| **Please describe:** | Click here to enter text. | |  |

| 1. **Other comments?** | All procedures in this study are well established and routinely used by our research group |
| --- | --- |

**Attachments Checklist - Please attach the following if applicable:**

| **Ethics approval from non-UWA HREC** |  |
| --- | --- |
| **Full medical research protocol and/or Student Research proposal** |  |
| **Recruitment material** (e.g. advertisements, posters, flyers) |  |
| **Participant Information Form (PIF)** |  |
| **Participant Consent Form (PCF)** |  |
| **Additional PIF and PCF for parent, teacher, school, as needed** |  |
| **Questionnaire / survey instrument** |  |
| **Other docs** (e.g. contracts, agreements, focus group docs, detailed procedure info) |  |
| **Translations, where languages other than English are used above.** |  |
| **Related Approval & supporting documents** |  |

**Certification / signatures**

| **Chief Investigator or Supervisor of Higher Degree Research Student:** | I declare that:   - The information provided in this application is truthful and as complete as possible. - I undertake to conduct the research in accordance with the approved protocol, the [*National Statement on Ethical Conduct in Human Research*](http://www.nhmrc.gov.au/guidelines/publications/e72), 2007, relevant legislation and the policies and procedures of The University of Western Australia. - Where I am the Project Supervisor for research described to be conducted by a student of The University of Western Australia, I declare that I have provided guidance to the student in the design, methodology and consideration of ethical issues of the proposed research; that the student has received the relevant research and ethics training for this project; and, that I will monitor the project during data collection. - I make this application on the basis that the information it contains is confidential and will be used by The University of Western Australia for the purposes of ethical review and monitoring of the research project described herein, and to satisfy reporting requirements to regulatory bodies. The information will not be used for any other purpose without my prior consent.   **Signed: danny.green@uwa.edu.au**  (see comment below about using UWA email instead of ink signatures) | | | |
| --- | --- | --- | --- | --- |
|  | Name: | Winthrop Professor Daniel J Green | Date: | 21-Jul-14 |

| **Head of School Declaration:** | (*Where the Head of School or nominee has a conflict of interest with the proposed research, e.g. an investigator on the project, a member of the research group, or a personal relationship to any member of the research team, this Declaration is to be completed by the Deputy Head of School.*)  I declare that:   - I am satisfied that an adequate peer review has been conducted and that the research proposal is ready for submission for ethics approval. - The resources required to undertake this project are available. - The researchers have the skill and expertise to undertake this project appropriately.   **Signed: tim.ackland@uwa.edu.au**  (see comment below about using UWA email instead of ink signatures) | | | |
| --- | --- | --- | --- | --- |
|  | Name: | Timothy Ackland | Date: | 21-Jul-14 |

| UWA policy deems this document as signed, if you send it attached to an email **from your UWA email address**. Alternatively you can sign the signature page, scan that page and send it with the application documents. |
| --- |
| For example, the form can be filled out by the CI, emailed as an attachment to the Head of School, and then **Forward**ed (to ensure all attachments and email from/to/date lines are carried forward) to the Human Research Ethics Office (HREO) at [hreo-research@uwa.edu.au](mailto:hreo-research@uwa.edu.au) – this method avoids the need for ink signatures, preserves the best available viewing and search quality, saves paper and saves time. |
